# Case-control study of behavior data from automated milk feeders in healthy or diseased dairy calves

**DOI:** 10.3168/jdsc.2021-0153

**Published:** 2022-03-03

**Authors:** Jannelle L. Morrison, Charlotte B. Winder, Catalina Medrano-Galarza, Pauline Denis, Derek Haley, Stephen J. LeBlanc, Joao Costa, Michael Steele, David L. Renaud

**Affiliations:** 1 Department of Population Medicine, University of Guelph, Guelph, ON, Canada, N1G 2W1; 2 Programa de Especialización en Bienestar Animal y Etología, Facultad de Medicina Veterinaria, Fundación Universitaria Agraria de Colombia, Bogotá, Colombia, Cll170#54a-10; 3 Université Clermont Auvergne, INRAE, VetAgro Sup, UMR Herbivores, 63122 Saint-Genès-Champanelle, France; 4 Department of Animal and Food Science, University of Kentucky, Lexington 40506; 5 Department of Animal Biosciences, Animal Science and Nutrition, University of Guelph, Guelph, ON, Canada N1G 1Y2

## Abstract

Group housing of preweaning dairy calves is increasing in popularity throughout the dairy industry. However, it can be more difficult to individually monitor calves to identify disease in these group systems. Automated milk feeders (AMF) not only provide producers with the opportunity to increase the milk allowance offered to preweaning calves but they can also monitor individual feeding behaviors that could identify calves at increased risk of disease. The objective of this retrospective case-control study was to determine how feeding behaviors change in preweaning calves leading up to and during a disease bout. This study was conducted between fall 2015 and fall 2016 on 2 commercial dairy farms in Ontario, Canada. Producers' treatment records for respiratory or enteric illness were used to identify cases. Control calves were selected from calves not treated for disease and matched on the days on the AMF. Both farms housed calves in dynamic groups of 9 to 11 calves with an AMF and fed milk replacer. Differences in feeding behaviors, including milk consumption, drinking speed, rewarded visits, unrewarded visits, and total visits to the AMF per day, were analyzed by mixed models accounting for repeated measures. Data were analyzed for the 7 d before, the day of, and 7 d after treatment. A total of 28 cases and 28 control calves (n = 56) were analyzed. Calves with disease consumed significantly less milk than their healthy counterparts, beginning 5 d before disease and until 3 d after disease detection. Sick calves had fewer unrewarded visits starting 3 d before until 2 d after illness detection. Sick calves drank significantly more slowly starting 4 d before illness detection until the day after illness detection compared with healthy controls. No differences were found between cases and controls for rewarded visits. Calves on a high plane of milk nutrition significantly alter feeding behaviors before illness detection. Data from AMF on feeding behaviors may help to detect disease earlier in preweaning dairy calves.

## Highlights

• Automated milk feeders have the potential to aid producers in disease detection.
• Milk consumption detected disease 5 d before disease detection by the producer.
• Drinking speed detected disease 4 d before disease detection by the producer.
• Unrewarded visits to the feeder detected disease 3 d before disease detection by the producer.
• Rewarded visits to the feeder were not useful in detection of disease by the producer.

## Graphical Abstract

**Figure:**
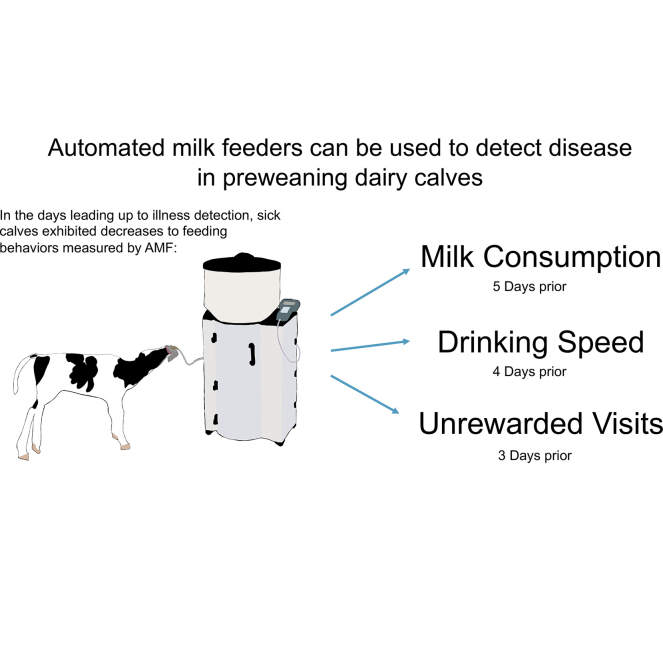
**Summary:** Automated milk feeders are computerized systems that provide producers with the opportunity to increase the milk allowance offered to preweaning calves. These computerized systems are thought to potentially aid in disease detection of preweaning dairy calves using individual feeding metrics. This retrospective case-control study analyzed data from 56 preweaning dairy calves (28 cases and 28 controls) and found milk consumption and drinking speed to be valuable disease predictors as early as 5 and 4 days before disease diagnosis, respectively. Unrewarded visits were found to be a useful predictor 3 days before disease diagnosis. Rewarded visits were not found to be a useful disease predictor.

Preweaning dairy calves are traditionally housed individually to decrease calf-to-calf transmission of pathogens (Jorgensen et al., 2017). However, group housing of preweaning calves has increased in popularity because of the perceived welfare benefits, lower labor requirements, and changes in the public perception of individual housing (Rushen et al., 2008; Costa et al., 2016; Perttu et al., 2020). In a Canadian survey completed in 2015, 34% of dairy producers reported group housing of preweaning dairy calves (Winder et al., 2018), which increased from 12% reported in a 2007 survey completed in Québec, Canada (Vasseur et al., 2010). Group housing can benefit calf welfare by allowing for social interaction and an increased ability to display natural behaviors (Costa et al., 2016). Group housing also improves the transition to solid feed, leading to increased postweaning growth (de Paula Vieira et al., 2010; Costa et al., 2015). However, group housing can make it more difficult to monitor each calf individually, which could lead to reduced disease detection (Cramer et al., 2020).

To feed calves in groups, an option available to producers is an automated milk feeding system (**AMF**). These computerized systems can decrease labor costs, allow producers to provide an elevated plane of milk nutrition (Kack and Ziemerink, 2010), and can provide data for each calf's feeding behavior. Monitoring these individual feeding behaviors is thought to aid in disease detection in preweaning calves (Costa et al., 2020). Previous studies have identified decreased milk consumption and drinking speed (Johnston et al., 2016; Knauer et al., 2017; Cramer et al., 2020), and changes to rewarded (Knauer et al., 2017; Lowe et al., 2020), unrewarded (Svensson and Jensen, 2007; Johnston et al., 2016; Knauer et al., 2017), and total visits to the AMF (Borderas et al., 2009; Lowe et al., 2020) as potential disease predictors. However, there have been inconsistent associations between these behaviors and disease. For example, Borderas et al. (2009) found a decrease in total visits to the AMF for sick calves, whereas Lowe et al. (2020) found an increase in total visits. Differences in the age of calves at illness detection, daily milk allowance, number of calves per pen, and number of days a calf has been on the AMF may also affect the ability of these metrics to detect disease. The literature on AMF data to identify or detect morbidity in calves is limited and inconsistent. A recently completed scoping review identified only 13 published articles on the topic, indicating a need for more primary research in this area (Morrison et al., 2021). Therefore, the objective of this retrospective case-control study was to assess changes in feeding behavior exhibited by preweaning calves before and during an illness event. We hypothesized that sick calves would consume less milk, drink milk at a slower rate, and visit the feeder less often than their healthy counterparts.

This retrospective case-control study was approved by the University of Guelph Animal Care Committee (Animal Use Protocol #3212). This study is reported following the STROBE-Vet reporting guidelines for observational studies (O'Connor et al., 2016; Sargeant et al., 2016). The data from this study were previously collected as a part of a larger study completed in Ontario, Canada (Medrano-Galarza et al., 2017). Two dairy farms were selected based on their size, use of an AMF, proximity to the University of Guelph, and willingness to participate in this study. Farm 1 had a herd size of 120 milking cows and farm 2 had 85 milking cows. Both farms housed milk cows in a freestall system and were visited 4 times, once each season between fall 2015 (September 21) and fall 2016 (September 21) to collect treatment records, birth records, and AMF data for any calf born during the trial period. Fall visits occurred between November 2 and December 1, 2015; winter visits between February 3 and March 16, 2016; spring visits between April 19 and May 30, 2016; and summer visits between August 3 and September 6, 2016. On farm 1, calves were introduced to the group pen between 6 and 9 d of age and had an average of 11 calves per pen; on farm 2, calves were introduced on the day of birth to a pen with 2 calves, and then moved at 4 d of age to a larger pen with an average of 9 calves per group. Both farms group housed calves with an AMF (CF1000, Förster-Technik) and used milk replacer to feed preweaning calves. Farm 1 fed calves 9 L/d of milk replacer for the first 46 d of life, and farm 2 used a step-up milk feeding program for the first 17 d, starting from 6 L/d and then allowing calves to drink 10 L/d until d 46. Both farms had one AMF feeder station for each pen of calves.

During farm visits, birth records and treatment records for all calves were obtained. For the purpose of this study, treatment records were used as proxies for disease events. On both farms, producers used similar methods to identify sick calves. Both used a physical check to evaluate the attitude of the calf, an elevated body temperature (≥39.5°C), and potential signs of respiratory (eye or nose discharge, coughing) or enteric disease. On farm 1, neonatal calf diarrhea (**NCD**) was treated with injections of an antibiotic (trimethoprim-sulfadoxine, Trimidox; Vetoquinol N.-A. Inc.) and an anti-inflammatory drug [meloxicam, Metacam; Boehringer Ingelheim (Canada) Ltd.], whereas bovine respiratory disease (**BRD**) was treated with an antibiotic—either florfenicol combined with flunixin (Resflor; Merck Animal Health) or tulathromycin (Draxxin; Zoetis Inc.). On farm 2, NCD was treated with an oral antimicrobial (Scour Plug; neomycin sulfate and sulfamethazine; Can-Vet Animal Health Supplies) and BRD was treated predominantly with florfenicol (Nuflor; Merck Animal Health) and meloxicam [Metacam; Boehringer Ingelheim (Canada) Ltd.].

During each farm visit, treatment records were obtained and calves that were treated for either NCD or BRD were eligible to be chosen as cases. Calves were eligible to be chosen as cases until the beginning of weaning and were only selected as a case once. Cases could have been treated for either NCD or BRD, and day of treatment was considered to be d 0. Healthy control calves were all selected at the end of the study period from calves that did not receive any treatment for disease. Control and case calves were matched by farm and to the same number of days on the AMF.

As a calf entered the AMF, a radiofrequency identification (**RFID**) tag in their ear was scanned and individual behavioral feeding metrics were recorded onto a computer. For each case and control calf, all information pertaining to milk feeding plan for the preweaning period was obtained from the AMF to aid in analyses. These data included type of milk fed to the calves (milk replacer), milk feeding regimen [starting milk allotment (L/d), peak milk allotment (L/d), latency to peak milk allotment and length of peak, maximum meal size, and minimum time between meals], weaning method (age at beginning of wean, length of weaning period (days)], and age when fully weaned. Individual feeding metrics for each case and control calf were exported from the AMF Calf Manager software for the 14 d surrounding a treatment event. Feeding metrics included daily milk consumption [amount of milk consumed by each calf per 24-h period (L/d)], drinking speed [the average speed at which a calf ingested all of its milk meals in a 24-h period (mL/min)], rewarded visits (the number of times a calf entered the AMF and received a milk meal each day), unrewarded visits (the number of times a calf entered the AMF and was not provided with a milk allotment), and total visits to the feeder (number of rewarded and unrewarded visits added together).

Calf treatment records and AMF data were imported into Excel (Microsoft Corp.) and cleaned and sorted. Excel files were then imported into STATA 15 (Stata/IC Version 15.1 for Mac, StataCorp) for analysis. Descriptive statistics were reviewed for all outcome variables. Mixed linear regression models accounting for repeated measures with an unstructured correlation structure were built to evaluate the association between case or control classification and each of the following outcomes: total daily milk consumption (L/d), average daily drinking speed (mL/min), rewarded visits (visits/d), unrewarded visits (visits/d), and total visits (visits/d). Case/control status and day relative to treatment were forced as fixed effects, and an interaction term between case/control status and day was also evaluated. When the interaction term had *P* < 0.05, contrast statements were evaluated to compare health status (case or control) by day to determine the effect between health status and day relative to treatment. Calf was used as a random effect to account for repeated measurement in each model. Model fit was assessed for each model using residuals. The assumptions of normality and homoscedasticity of best linear unbiased predictors (BLUPs) were assessed graphically for each model. All models except unrewarded visits met all required assumptions of model fit. Attempts were made to normalize the unrewarded visits model through logarithmic and quadratic transformations but neither improved the fit, so the data were analyzed nonparametrically using a nonparametric kernel regression model. To determine sensitivity and specificity of each feeding behavior on disease prediction, optimal cut-points were determined separately for each variable using Youden's index.

A total of 28 cases and 28 controls (n = 56 calves) were enrolled in the study. On treatment day (d 0), healthy (control) calves were a mean (±SD) of 29 ± 11 d of age, and sick (case) calves were 25 ± 11 d of age. Farm 1 provided 19 cases (8 enteric disease, 11 respiratory disease) and 19 controls. Farm 2 provided 9 cases (2 enteric disease, 7 respiratory) and 9 controls. Healthy calves that were utilized more than once as a control were removed from the analysis. On both farms, herd-level disease prevalence was calculated using producer treatment records (for morbidity and mortality) for calves born and kept during the trial period (September 22, 2015, to September 21, 2016). On farm 1, a total of 53 heifer calves were born over the trial period; of those calves, 27 were never treated, 2 were treated for both respiratory and enteric disease, 13 were treated for enteric disease, and 11 were treated for respiratory disease. This led to a disease prevalence of 49.1% for farm 1. On farm 2, a total of 26 heifer calves were born during the trial period; of those calves, 8 were never treated, 1 calf was treated for both respiratory and enteric disease, 2 calves were treated for enteric disease, and 15 calves were treated for respiratory disease. This led to an on-farm disease prevalence of 34.6%.

Milk consumption was significantly lower in sick calves (*P* < 0.001), and the interaction between health status and day was significant (*P* < 0.001). Sick calves (cases) drank less milk than controls on d 5(−1.23 L/d; 95% CI: −2.23 to −0.23; *P* = 0.016), d 4 (−1.62 L/d; 95% CI: −2.60 to −0.63; *P* = 0.001), d 3 (−1.97 L/d; 95% CI: −2.96 to −0.99; *P* **<** 0.001), d 2 (−2.24 L/d; 95% CI: −3.23 to −1.26 *P* **<** 0.001), and d 1 (−2.80 L/d; 95% CI: −3.79 to −1.81; *P* **<** 0.001) before producer treatment compared with control calves (Figure 1). On the day of diagnosis, sick calves drank 2.16 L/d (95% CI: −3.14 to −1.17; *P* < 0.001) less than control calves (Figure 1). Milk consumption (cut-point 6.65 L/d) had a sensitivity of 71.43% and a specificity of 57.14% for the detection of disease on the day of producer treatment.

**Figure 1:**
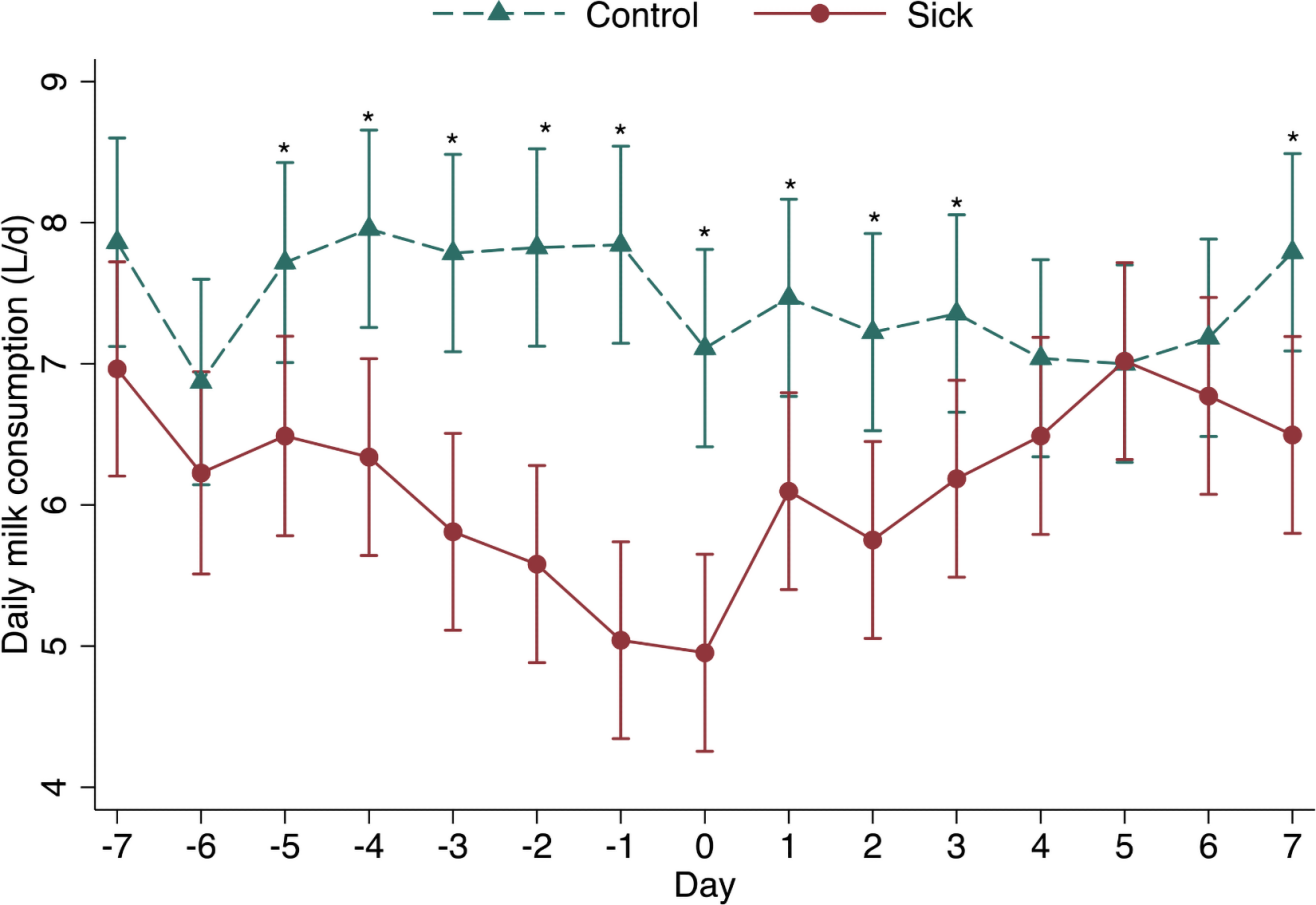
Model-predicted differences in daily milk consumption in the 7 d before, 7 d following, and on the day of disease treatment (day 0) for 28 case and 28 control calves aged 27 d ± 11 d (mean ± SD) housed on 2 commercial dairy farms in Ontario. *Significant difference: *P* < 0.05.

Drinking speed was significantly slower in sick calves (*P* = 0.0049), and the interaction term between health status and day was significant (*P* = 0.002). Sick calves drank more slowly than control calves on d 4 (−129.70 mL/min; 95% CI: −237.65 to −21.78; *P* = 0.018), d 3 (−179.31 mL/min; 95% CI: −287.24 to −71.40; *P* = 0.001), d 2 (−117.93 mL/min; 95% CI: −225.85 to −10.01; *P* = 0.032), and d 1 (−163.75 mL/min; 95% CI: −271.67 to −55.83; *P* = 0.003) before producer treatment (Figure 2). On the day of disease diagnosis, sick calves drank 158.4 mL/min more slowly than control calves (95% CI: −266.30 to −50.45; *P* = 0.004; Figure 2). Drinking speed (cut-point 492.63 mL/d) had a sensitivity of 71.43% and a specificity of 64.29% for the detection of disease on the day of producer treatment.

**Figure 2:**
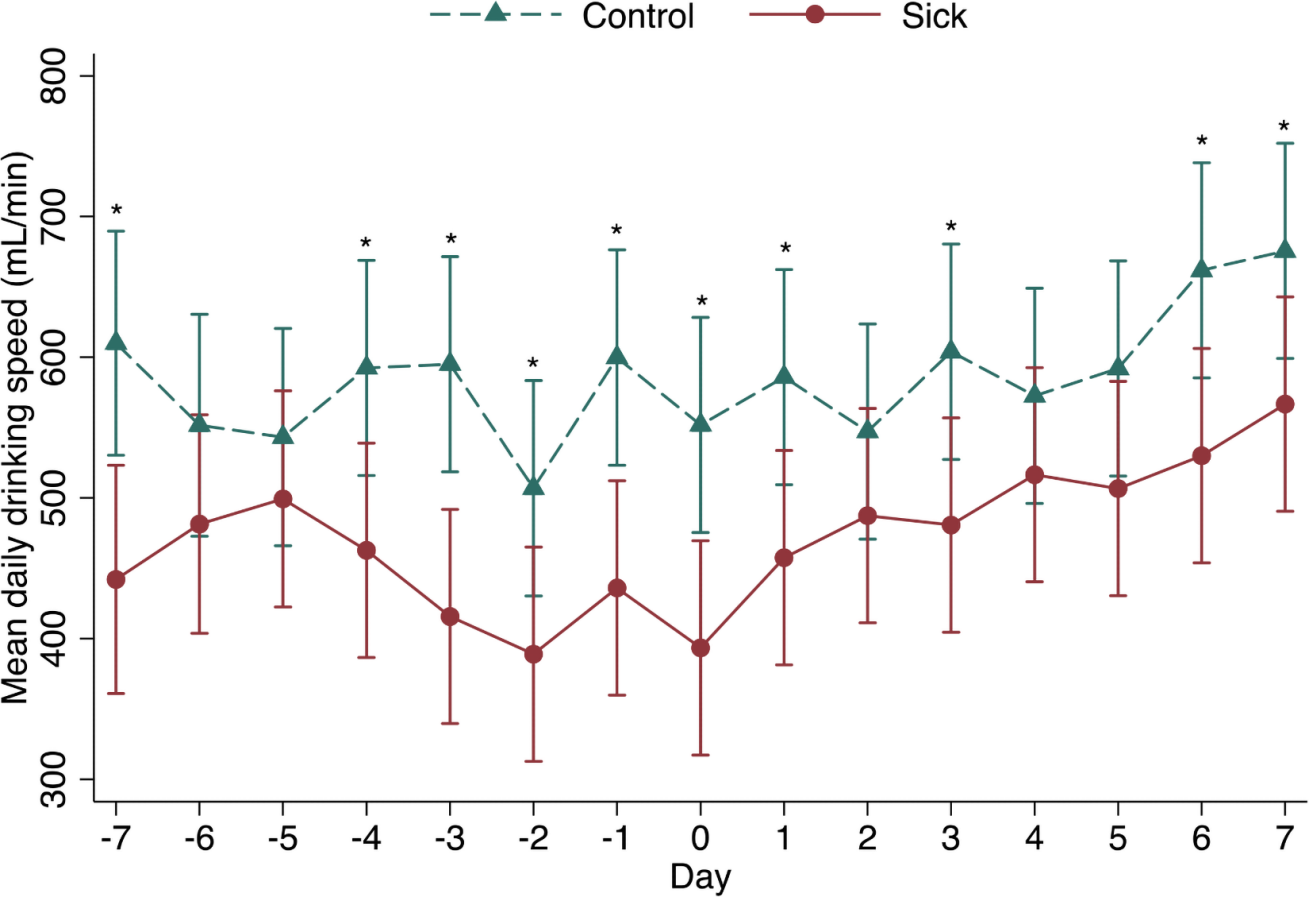
Model-predicted differences in daily drinking speed in the 7 d before, 7 d following, and on the day of disease treatment (day 0) for 28 case and 28 control calves with an average age of 27 d ± 11 d (mean ± SD) housed on 2 commercial Ontario dairy farms. *Significant difference: *P* < 0.05.

The number of unrewarded visits was significantly lower in sick calves compared with healthy control calves (95% CI: −0.05 to −0.03; *P* = 0.001; Figure 3). When looking at specific days before producer treatment, sick calves had fewer unrewarded visits on d 7 (−2.276 visits/d; *P* = 0.0228), d 5 (−2.362 visits/d; *P* = 0.0182), d 3 (−3.236 visits/d; *P* = 0.0012), d 2 (−2.613 visits/d; *P* = 0.0090), and d 1 (−4.102 visits/d; *P* < 0.0001) before producer treatment, as well as on producer treatment day (−2.200 visits/d; *P* = 0.0278). No differences were found with respect to unrewarded visits on d 6 or d 4 before producer treatment (Figure 3). Following producer treatment, sick calves had significantly fewer unrewarded visits to the feeder on d 2, 4, 5, and 6 compared with healthy control calves (Figure 3). Unrewarded visits (cut-point 0.5 visits/d) had a sensitivity of 75.00% and a specificity of 50.00% for the detection of disease on the day of producer treatment.

**Figure 3:**
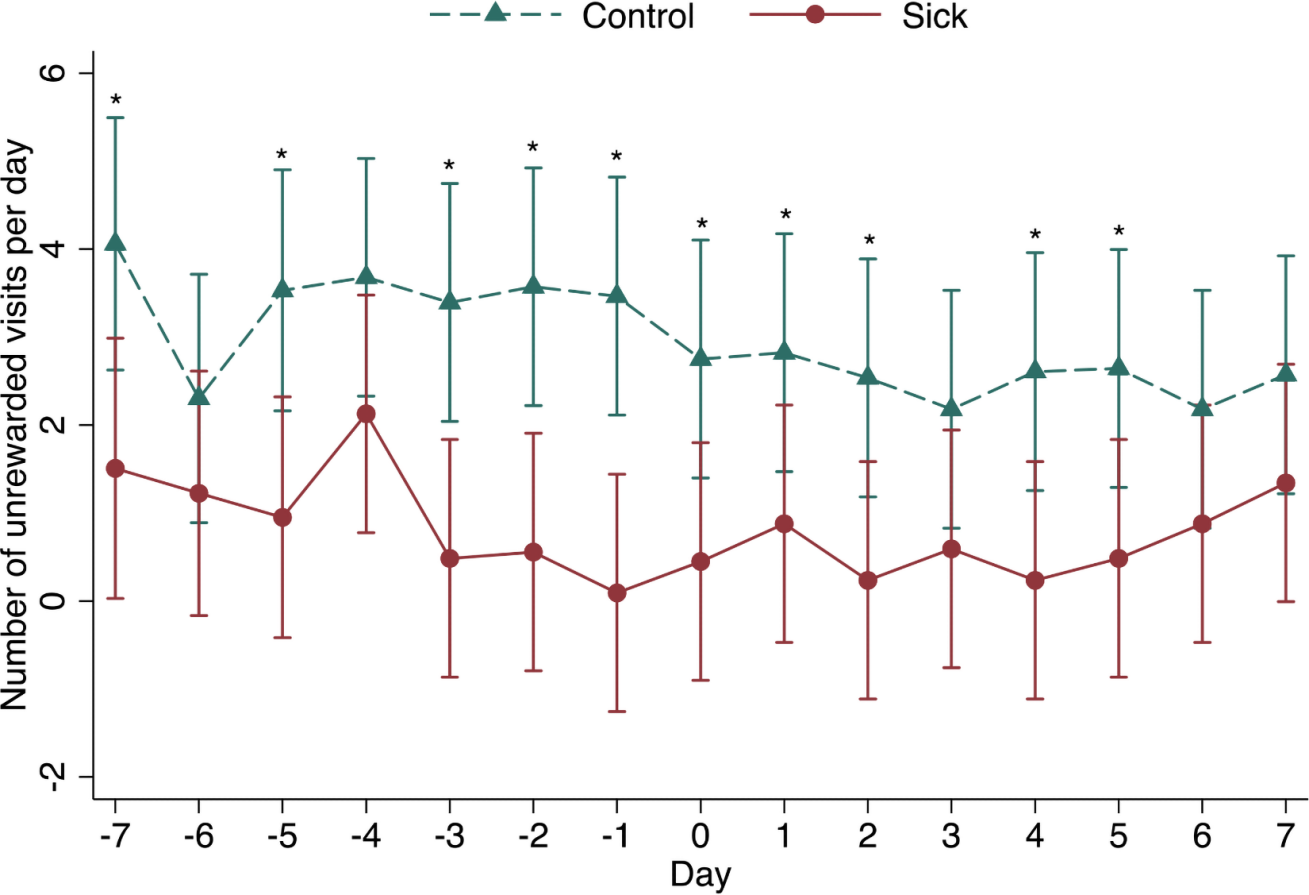
Model-predicted differences in unrewarded visits in the 7 d before, 7 d following, and on the day of disease treatment (day 0) for 28 case and 28 control calves with an average age of 27 d ± 11 d (mean ± SD) housed on 2 commercial Ontario dairy farms. *Significant difference: *P* < 0.05.

Sickness behaviors occur through the interaction of pathogens and the calf's immune system (Johnson, 2002). The calf produces inflammatory cytokines, which circulate through the bloodstream, inducing a fever and sickness behaviors such as anorexia, lethargy, and general disinterest in socialization (Johnson, 2002). Although these behaviors are more difficult to detect in group-housed calves (Johnson, 2002; Cramer et al., 2020), AMF data can help with disease detection (Svensson and Jensen, 2007; Borderas et al., 2009; Knauer et al., 2017). Our results showed that milk consumption, the frequency of unrewarded visits, and drinking speed differed before detection of illness by producers. Similar results were found by Knauer et al. (2017), who observed that drinking speed, milk consumption, and unrewarded visits were lower up to 4 d before illness detection by producers.

Daily milk allotment has been shown to affect the utility of feeding behaviors as suitable predictors for disease. Calves on a high milk allowance (12 L/d or ad libitum) decreased milk consumption on the day of illness detection compared with healthy calves (Borderas et al., 2009; Swartz et al., 2017; Lowe et al., 2020), whereas calves on restricted milk allowances (5 to 8 L/d) showed no difference in daily milk consumption during a disease event (Svensson and Jensen, 2007). Our findings are similar to studies that provided a high milk allowance, even though calves in our study had a daily milk allowance of 9 to 10 L. This suggests that even at a moderate milk feeding plane, differences in milk consumption can be detected before illness detection.

Unrewarded visits to the AMF have been shown to be a useful predictor in disease detection in preweaning dairy calves. Our results are consistent with Knauer et al. (2017), who found a decrease in unrewarded visits that started 4 d before illness detection. Sutherland et al. (2018) also found that ad libitum-fed calves had significantly fewer unrewarded visits starting 2 d before diarrhea diagnosis compared with healthy control calves. Rewarded visits were not different between cases and controls. This suggests that even when illness is present, under the study conditions, hunger may be a stronger motivation than sickness behaviors. The calf will still visit the AMF to feed but may drink at a slower rate and not visit the feeder more than necessary. Our results agree with those of other studies (Svensson and Jensen., 2007; Swartz et al., 2017; Sutherland et al., 2018) that reported rewarded visits to be not useful in detecting disease. It is interesting to note that there was variation in daily milk allowance, with our study feeding 9 to 10 L/d, Swartz et al. (2017) feeding 12 L/d, Sutherland et al. (2018) feeding 6 L/d, and Svensson and Jensen (2007) feeding 5.6 to 7.2 L/d. This provides evidence that regardless of daily milk allotment, rewarded visits may not be a useful feeding behavior in detecting disease, as sick calves are still motivated to drink; however, they will visit the feeder only when necessary, as observed in our decrease in unrewarded visits.

One limitation of this study was that health status was determined using producer treatment records. This may not be as accurate as daily health scoring in detecting sick calves. It is known that producer records are not always reliable and accurate for disease detection, and therefore may not be the best diagnostic tool (Vasseur et al., 2012). Many producers wait until there are visible signs of disease present to treat a sick calf; however, the disease may be present at the subclinical level days before it is noticed by the producer. Studies have shown that producers often under- or over-diagnose disease in preweaning dairy calves, which may have affected our results. Gulliksen et al. (2009) found that the most reliable producers in Norway underestimated disease up to 40% in preweaning calves. Olson et al. (2019) compared clinical health scoring to producer treatment records and found that a high proportion of calves treated for enteric disease or dehydration did not show clinical signs of illness. Furthermore, when thoracic ultrasound was used to identify diseased calves, 28% of treated calves showed no signs of thoracic lung lesions (Olson et al., 2019). Despite this limitation, this study highlights that drinking behaviors could be used to identify calves that require additional attention or examination, as many of the behaviors changed several days before disease treatment.

The results of this retrospective case-control study provide further evidence that sick calves on an AMF alter several aspects of their feeding behaviors leading up to a disease event. We found that sick calves drank more slowly and consumed less milk up to 4 and 5 d, respectively, before initiation of treatment by the producer. Sick calves also had fewer unrewarded visits to the AMF 3 d before illness detection by producer. However, our study was limited by the use of producer health records, which are less accurate than systematic clinical health scoring.

## References

[bib1] Borderas T.F., Rushen J., von Keyserlingk M.A.G., de Passille A.M.B. (2009). Automated measurement of changes in feeding behavior of milk-fed calves associated with illness. J. Dairy Sci..

[bib2] Costa J.H., Cantor M.C., Neave H.W. (2021). Symposium review: Precision technologies for dairy calves and management applications. J. Dairy Sci..

[bib3] Costa J.H.C., Meagher R.K., Von Keyserlingk M.A.G., Weary D.M. (2015). Early pair housing increases solid feed intake and weight gains in dairy calves. J. Dairy Sci..

[bib4] Costa J.H.C., von Keyserlingk M.A.G., Weary D.M. (2016). Invited review: Effects of group housing of dairy calves on behavior, cognition, performance, and health. J. Dairy Sci..

[bib5] Cramer C., Proudfoot K., Ollivett T. (2020). Automated feeding behaviors associated with subclinical respiratory disease in preweaned dairy calves. Animals (Basel).

[bib6] de Paula Vieira A., Von Keyserlingk M.A.G., Weary D.M. (2010). Effects of pair versus single housing on performance and behavior of dairy calves before and after weaning from milk. J. Dairy Sci..

[bib7] Gulliksen S.M., Lie K.I., Østerås O. (2009). Calf health monitoring in Norwegian dairy herds. J. Dairy Sci..

[bib8] Johnson R.W. (2002). The concept of sickness behavior: A brief chronological account of four key discoveries. Vet. Immunol. Immunopathol..

[bib9] Johnston D., Kenny D.A., McGee M., Waters S.M., Kelly A.K., Earley B. (2016). Electronic feeding behavioural data as indicators of health status in dairy calves. Ir. J. Agric. Food Res..

[bib10] Jorgensen M.W., Janni K., Adams-Progar A., Chester-Jones H., Salfer J.A., Endres M.I. (2017). Housing and management characteristics of calf automated feeding systems in the Upper Midwest of the United States. J. Dairy Sci..

[bib11] Kack M., Ziemerink J. (2010). Proc. First North American Conference on Precision Dairy Management 2010, Toronto, Canada.

[bib12] Knauer W.A., Godden S.M., Dietrich A., James R.E. (2017). The association between daily average feeding behaviors and morbidity in automatically fed group-housed preweaned dairy calves. J. Dairy Sci..

[bib13] Lowe G.L., Sutherland M.A., Waas J.R., Cox N.R., Schaefer A.L., Stewart M. (2021). Effect of milk allowance on the suitability of automated behavioral and physiological measures as early disease indicators in calves. Appl. Anim. Behav. Sci..

[bib14] Medrano-Galarza C., LeBlanc S.J., DeVries T.J., Jones-Bitton A., Rushen J., de Passillé A.M., Haley D.B. (2017). A survey of dairy calf management practices among farms using manual and automated milk feeding systems in Canada. J. Dairy Sci..

[bib15] Morrison J., Renaud D.L., Churchill K.J., Costa J.H.C., Steele M.A., Winder C.B. (2021). Predicting morbidity and mortality using automated milk feeders: A scoping review. J. Dairy Sci..

[bib16] O'Connor A.M., Sargeant J.M., Dohoo I.R., Erb H.N., Cevallos M., Egger M., Ersbøll A.K., Martin S.W., Nielsen L.R., Pearl D.L., Pfeiffer D.U., Sanchez J., Torrence M.E., Vigre H., Waldner C., Ward M.P. (2016). Explanation and elaboration document for the STROBE-Vet Statement: Strengthening the Reporting of Observational Studies in Epidemiology–Veterinary Extension. Zoonoses Public Health.

[bib17] Olson A., Sischo W.M., Berge A.C.B., Adams-Progar A., Moore D.A. (2019). A retrospective cohort study comparing dairy calf treatment decisions by farm personnel with veterinary observations of clinical signs. J. Dairy Sci..

[bib18] Perttu R.K., Ventura B.A., Endres M.I. (2020). Youth and adult public views of dairy calf housing options. J. Dairy Sci..

[bib19] Rushen J., de Passillé A.M., von Keyserlingk M.A.G., Weary D.M., Phillips C. (2008). The Welfare of Cattle.

[bib20] Sargeant J.M., O'Connor A.M., Dohoo I.R., Erb H.N., Cevallos M., Egger M., Ersbøll A.K., Martin S.W., Nielsen L.R., Pearl D.L., Pfeiffer D.U., Sanchez J., Torrence M.E., Vigre H., Waldner C., Ward M.P. (2016). Methods and processes of developing the Strengthening the Reporting of Observational Studies in Epidemiology—Veterinary (STROBE-Vet) Statement. Zoonoses Public Health.

[bib21] Sutherland M.A., Lowe G.L., Huddart F.J., Waas J.R., Stewart M. (2018). Measurement of dairy calf behavior prior to onset of clinical disease and in response to disbudding using automated calf feeders and accelerometers. J. Dairy Sci..

[bib22] Svensson C., Jensen M.B. (2007). Short communication: Identification of diseased calves by use of data from automatic milk feeders. J. Dairy Sci..

[bib23] Swartz T.H., Findlay A.N., Petersson-Wolfe C.S. (2017). Automated detection of behavioral changes from respiratory disease in pre-weaned calves. J. Dairy Sci..

[bib24] Vasseur E., Borderas F., Cue R.I., Lefebvre D., Pellerin D., Rushen J., Wade K.M., de Passille A.M. (2010). A survey of dairy calf management practices in Canada that affect animal welfare. J. Dairy Sci..

[bib25] Vasseur E., Pellerin D., de Passillé A.M., Winckler C., Lensink B.J., Knierim U., Rushen J. (2012). Assessing the welfare of dairy calves: Outcome-based measures of calf health versus input-based measures of the use of risky management practices. Anim. Welf..

[bib26] Winder C.B., Bauman C.A., Duffield T.F., Barkema H.W., Keefe G.P., Dubuc J., Uehlinger F., Kelton D.F. (2018). Canadian National Dairy Study: Heifer calf management. J. Dairy Sci..

